# Production, medium optimization, and structural characterization of an extracellular polysaccharide produced by *Rhodotorula minuta ATCC 10658*

**DOI:** 10.1002/fsn3.1792

**Published:** 2020-07-19

**Authors:** Hamid Reza Samadlouie, Kambiz Jahanbin, Parisa jalali

**Affiliations:** ^1^ Department of Food Science and Technology Faculty of Agriculture Shahrood University of Technology Shahrood Iran

**Keywords:** extracellular polysaccharide, response surface methodology, *Rhodotorula minuta*, structural study

## Abstract

Several strains of microorganism are capable of converting carbohydrates into extracellular polysaccharide. The preset research is a first effort made to optimize extracellular polysaccharide (CRMEP) by *Rhodotorula minuta* ATCC 10658 using one factor at time and response surface methods. One factor at time was applied in the initial screening of substrates prior to optimization study. Of all the substrates examined, starch as carbon source and defatted soy bean powder as protein source were discovered to be best for CRMEP production. Response surface analysis revealed that 15 g/L starch and 30g/L defatted soy bean powder were the optimal chemical conditions. The model predicted 13.22 g/L for CRMEP, which went along with the experimentally observed result. Purification of CRMEP by anion‐exchange column of DEAE‐cellulose yielded RMEP. Structural investigation indicated that the main chain of RMEP was composed of (1 → 3) and (1 → 4)‐linked mannopyranosyl residues, with branches attached to *O*‐6 of some (1 → 3)‐linked mannopyranosyl residues. The branches were composed of Glc*p*‐(1 → residues.

## Graphical abstract

Rhodotorula minuta ATCC 10658’ extracellular polysaccharide was optimized and characterized.

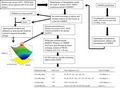

## 1 INTRODUCTION

Polysaccharides as indispensable biopolymers in all living organisms made of more than ten monosaccharides are linked together through sequential glycosidic bonds. Such valuable and ubiquitous biopolymers cover a wide array of biological properties like antioxidant (Shabani, Askari, Jahanbin, & Khodaeian, 2016; Siu, Xu, Chen, & Wu, 2016), antitumor (Meng, Liang, & Luo, 2016), anti‐inflammatory (Lajili, Deghrigue, Amor, Muller, & Bouraoui, 2016), and immunomodulatory activities (Yang, Jia, Meng, Wu, & Mei, 2006). Polysaccharides could be definitely found in plants, animals, and microorganisms. The preferred features for microbial extracellular polysaccharides (EPS) are as follows: high EPS yield, high EPS productivity, capability to grow in sufficiently low‐cost substrates (Villano et al., 2014) and curative properties (Galinari et al., 2017; Kogani et al., 2008; Sutherland, 2001). As far as EPS productions are concerned, microbial strains have been extensively examined (Lu et al., 2019; Ma, Mao, Geng, Wang, & Xu, 2013). Rhodotorula, belonged to environmental basidiomycetous, is a genus of unicellular pigmented yeasts. Importantly, three out of 46 species of the genus, namely *R. mucilaginosa, R. glutinis,* and *R. minuta*, have been rarely recognized as human pathogens (Arendrup et al., 2014). *R. minuta* has been recognized as a well‐known source of EPS served in food, cosmetic, and pharmaceutical fields (Seveiri et al., 2019). The influence of substrate properties and their concentrations on microbial productions has been well‐characterized. The quality and quantity of carbon and nitrogen in medium highly impact on the microbial proliferation and EPS synthesis (Kim et al., 2003; López et al., 2003; Nicolaus, Kambourova, & Oner, 2010). Organic carbon and nitrogen sources support microbial growth rate and EPS production (Czaczyk & Wojciechowska, 2003; Görke & Stülke, 2008). The synthesis of heteroglycan made of D‐glucose, D‐mannose, and D‐glucuroic acid was stimulated by organic nitrogen (Elinov et al., 1988). Given this in view, enhanced productivity can be achieved by using proper substrates and optimization methods (Ma et al., 2013). Among various statistical methods, response surface methodology (RSM) has worldwide served as a pioneer in mathematical analysis of the variable factors on responses. Such a method was served to optimize the valuable products produced by fermentation method (Ma et al., 2013; Malinowska, Krzyczkowski, Łapienis, & Herold, 2009). Prior to the optimization, screening method like one a factor at a time method was served to identify the key substrates among the various selected substrates (Singh, Singh, Tripathi, Khare, & Garg, 2011).

*Rhodotorula minuta (R. minuta*) capability of producing EPS has been well‐documented (Ramirez, 2016; Seveiri et al., 2019). However, there are no available data for statistical optimization of EPS by *R. minuta* in submerge conditions. More importantly, the structural features of *R. minuta´* EPS have not been well‐characterized yet. Therefore, the objective of this research was the optimization of *R. minuta*’ EPS production, purification of the produced EPS by anion‐exchange column chromatography and finally, characterization of the main purified EPS by gas chromatography‐mass spectrometry (GC–MS).

## 2 MATERIALS AND METHODS

### 2.1 Materials and chemicals

*Rhdotorula minuta* ATCC 10658 was purchased from Persian Type Culture Collection (PTCC). DEAE‐Cellulose A52 and bovine serum albumin (BSA) were purchased from Pharmacia Co. and Merck, respectively. All materials were also provided from Merck. Aqueous solutions were prepared with ultra‐pure water from a Milli‐Q water purification system (Millipore). All other reagents used in this study were of analytical grades.

### 2.2 General methods

Concentrations were performed under reduced pressure in a rotary evaporator (Heidolph Laborota 4,000 efficient rotary evaporator, Germany). The products were dried by vacuum freeze‐drying (Christ Alpha 1–2 freeze‐dryer). Protein in the exopolysaccharide was quantified according to the Bradford method (Bradford, 1976), using BSA as the standard. Ultraviolet‐visible absorption spectra were recorded with a VarianCary100‐Bio UV/visible spectrophotometer. Gas chromatography‐mass spectrometry (GC–MS) was done on a HP5890 (II) instrument (Hewlett‐Packard Component, USA) with an HPS quartz capillary column (25 m × 0.22 mm × 0.20 μm), and at temperatures programmed from 120 ºC (maintained for 2 min) to 260ºC (kept for 40 min) at a rate of 15ºC/min.

### 2.3 Microorganisms, inoculums, and cultivation conditions

The strain was grown on potato dextrose agar (PDA), and for long‐term storage, it was incubated at 4°C. 250 ml Erlenmeyer flasks with 50 ml medium was used for yeast growth. Seed cultures contained 20 g/L glucose and 10 g/L yeast extract with essential mineral elements. After sterilization, *R. minuta* ATCC 10658 was cultivated in seed medium and centrifuged cells were used in fermentation media.

### 2.4 Fermentation media

Various substrates such as carbon and protein resources as variable factors were utilized to examine EPS productions in the fermentation media. Mineral elements (magnesium sulfate (7 mg/L), calcium chloride (2 mg/L), di‐potassium hydrogen phosphate (1 g/L), ammonium sulfate (5/2 g/L), sodium chloride (0.1 g/L)), a rotary shaker at 180 RPM and 28°C were selected as constant factors. 3 percent of fresh seed culture was used in the fermentation media.

### 2.5 Isolation and purification of exopolysaccharide

Yeast cells were removed from the submerge medium by dilution with distilled water (two times) and centrifugation (12,000 g, 5 min). The cell‐bound exopolysaccharides were removed with 0.25 N NaOH for 2 hr and mixed with supernatant. The supernatant was concentrated 3‐fold with the rotary evaporator at 45°C. The concentrated solution was deproteinated by sevag method (1‐butanol: chloroform at a ratio of 1:4, v/v) (Staub, 1965). After the removal of sevag reagent, to remove small molecules, the solution was dialyzed against deionized water for 48 hr. The non‐dialyzate was then precipitated with 96% ethanol (1:4, v/v, stored for 24 hr at 4°C). Finally, the precipitate, collected by centrifugation, was lyophilized to give crude *R. minuta* ATCC 10658’ exopolysaccharide (CRMEP). The CRMEP was dissolved in deionized water and filtered (0.45 μm). The solution was passed through an anion‐exchange column of DEAE‐Cellulose A52 (2.6 × 30 cm) (Jahanbin, 2018). The elution was a gradient of 0–1 M aqueous solution of NaCl. The collected fractions were monitored by the phenol–sulfuric acid colorimetric method at 490 nm (Dubois, Gilles, Hamilton, Rebers, & Smith, F., 1956). Fractions, which corresponded to the major peak, were pooled, dialyzed, and lyophilized to result white pure polysaccharide (RMEP) and used for further study (Beigi & Jahanbin, 2019).

### 2.6 Structure determination of RMEP

Methylation analysis was employed to determine the positions of glycosidic linkages and their proportions in RMEP. RMEP was methylated according to the Needs and Selvendran method (Needs & Selvendran, 1993). Briefly, 1 ml DMSO was added to dry RMEP (5 mg) in a 25 ml flask. The mixture was sonicated at room temperature for 20 min and 4 ml methyl sulfinyl methyl sodium (MSMS) was added to the solution to form a gel, and the mixture was again treated by sonication for 20 min. 0.3 ml methyl iodide was then added, and the mixture was sonicated for 15 min at 25°C once more. After incubation for 6 hr at room temperature, excess MSMS was removed by the addition of water and subsequent centrifugation (Chaplin & Kennedy, 1994). The methylated polysaccharide was extracted with chloroform (4 ml) and was examined by IR spectrometry. Complete methylation was confirmed by the absence of an absorption peak related to the hydroxyl group in the region of 3200–3700 cm^‐1^. The methylated RMEP was hydrolyzed with formic acid and TFA (2 M), reduced with NaBD_4_ for 24 hr, and finally acetylated with acetic anhydride–pyridine (1:1). The partially methylated alditol acetates were analyzed by GC–MS (Sahragard & Jahanbin, 2017), according to the procedure above.

### 2.7 Statistical method

One factor at a time method was used to identify the key substrates among the various carbons and nitrogen sources which exerted high influence on EPS production, then the selected carbon and nitrogen sources were optimized using response surface method.

## 3 RESULTS AND DISCUSSION

### 3.1 Investigation of substrates on CRMEP production using one factor at time

#### 3.1.1 Carbon sources

**FIGURE 1:**
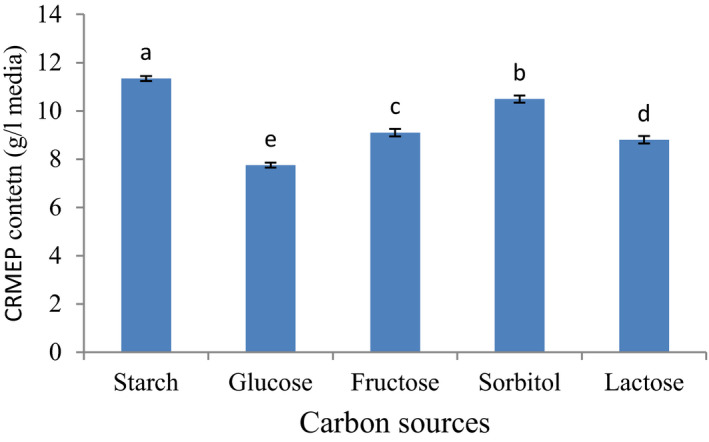
Effect of various carbon resources on CRMEP production

Temperature (21°C), yeast extract (20 g/L), pH (5.5), stirring round (180 rpm), minerals, and 4 days fermentation were constant factors. On the other hand, carbon sources (starch, glucose, fructose, sorbitol, and lactose) were variable factors. As can be seen in Figure 1, starch source was ranked as the best substrate for CRMEP production, followed closely by sorbitol. By contrast, glucose had the least impact on CRMEP production.

Many research put forward hypotheses that carbon sources as a main precursor for EPS production had the highest effect on the polysaccharide's properties and supported the high quantity of polysaccharide. Various research studies were carried out to investigate the effect of the carbon and nitrogen sources on polysaccharides production (Gientka, Bzducha‐Wróbel, Stasiak‐Różańska, Bednarska, & Błażejak, 2016; Khani et al., 2016). Khani et al., 2016 (Khani et al., 2016) stated that EPS production was stimulated by the high content of glucose. A study done by Gientka et al., 2016 (Gientka et al., 2016) revealed that, of all substrates used, carbon source significantly enhanced the yeast polysaccharide. Maalej et al., 2014 stated starch was discovered to be preferred over other carbon sources for EPS by *Pseudomonas stutzeri* AS22. Polysaccharide was better off than other carbon sources for ease of polymerization (Fan, Soccol, Pandey, & Soccol, 2007). However, microbial EPS depends upon the types of carbon source and the yeast species. Lactose, for instances, was ranked as the best substrate for EPS production by *Zunongwangia profunda* SM‐A87 (Liu et al., 2011), while Pavlova & Grigorova, 1999 (Pavlova & Grigorova, 1999) stated that sucrose was chosen to be the best source of carbon source for EPS by *Rhodotorula acheniorum MC*.

#### 3.1.2 Protein sources

**FIGURE 2:**
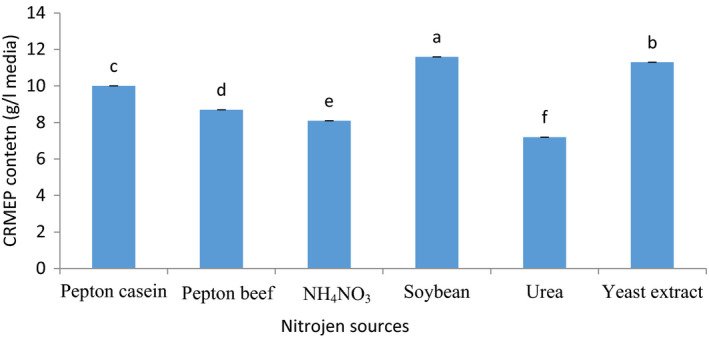
Effect of various nitrogen resources on CRMEP production

Temperature (21°C), starch (7 g/L), pH (5.5), stirring round 180 rpm, minerals, and the duration of 4 days fermentation were considered constant factors. The effects of nitrogen sources on CRMEP production were investigated. Inorganic nitrogen sources had a negligible impact on CRMEP yields (urea and NH_4_NO_3_). To be more precise, the least quantity of CRMEP was obtained in medium containing urea as nitrogen sources. However, organic nitrogen sources were rated as good substrates to stimulate *R. minuta ATCC 10658̕* CRMEP. Importantly, appreciable amount of CRMEP was achieved in medium containing soybean protein (Figure 2).

Based on the research conducted to evaluate EPS production, protein properties had a substantial impact on it. Liu et al., 2011 reported peptone stimulated *Zunonwangia profunda* to produce the higher content of EPS (8.90 g/L). Srinivas & Padma, 2014 stated that organic protein sources were more effective than inorganic nitrogen sources. *Cordyceps jiangxiensis JXPJ 0109 and Rhodotorula bacarum* were able to produce the highest content of EPS in media containing yeast extract and soybean protein, respectively (Jin et al., 2008; Shuangzhi & Zhenming, 2003).

### 3.2 Optimization of CRMEP production using RSM

Central Composite Rotatable Design (CCRD) was used as promising method for optimization. One factor at a time conducted disclosed that starch as carbon source and soybean as nitrogen sources were typically key factors enhancing CRMEP production by *R. minuta*. The data were statically analyzed using Design‐Expert software (6.0.10 version) and the method of the least squares was applied to formulate second equation. *R* ^2^ index was used as an adequacy of model (Giovanni, 1983). Significance at 5 percent level of confidence was selected to identify the effect of variables at linear, quadratic, and interactive level on response. ANOVAs results indicated that the model term and linear effect of starch and soybean powder having values “Prob > F” less than 0.05 were significant (Table 1).

**TABLE 1:** ANOVA for Response Surface Quadratic. Model Analysis of variance table

Source	Sum of Squares	*df*	Mean Square	*F* Value	*p*‐value Prob > F	
Model	43.09	5	8.62	206.23	<.0001	Significant
A‐starch	2.31	1	2.317	55.44	.0017	
B‐soya	32.64	1	32.64	781.01	<.0001	
AB	0.67	1	0.67	16.08	.0160	
A^2^	7.46	1	7.46	178.50	.0002	
B^2^	1.52	1	1.52	36.47	.0038	
Residual	0.16	4	0.04			
Lack of fit	0.16	3	0.05	10.81	.2191	not Significant
Pure error	0.005	1	0.005			
Core total	43.26	9				

As shown in Table 1, *F* value of soybean powder was higher than that of the carbon source; as a result, protein source was quantitatively more important than that of carbon source on CRMEP production. Starch and soybean powders were the variable factors as given in Table 1. Once, soybean concentration rose to 30 g/L along with the high and constant level of glucose (15 g/L), CRMEP production considerably climbed from 9.9 up to 13.2 (run 4 and 8). Similar patterns repeated at the lower rate for glucose substrate (run 4 and 10) (Table 2).

**TABLE 2:** Results of FCCCD using two variables indicating observed and predicted results

Run	Starch (g/L)	Soybean (g/L)	CRMEP (g/L media)	
			Observed	Predict
1	0 (12.5)	0 (22.5)	9.4	9.35
2	0 (12.5)	−1.4 (11.89)	7.80	7.64
3	0 (12.5)	0 (22.5)	9.3	9.35
4	1 (15)	−1 (15)	9.90	10.13
5	1.41 (16.4)	0 (22.5)	12.90	12.66
6	−1 (10)	1 (30)	13.14	13.09
7	−1.41 (8.96)	0 (22.5)	11.10	11.14
8	1 (15)	1 (30)	13.2	13.35
9	0 (12.5)	1.41 (33.11)	13.40	13.62
10	−1 (10)	−1 (15)	8.20	8.23

That is to say, soybean was more effective than starch on CRMEP production. Analysis of variance (ANOVA) was applied to fit a second order polynomial equation. The fitted equation of CRMEP production over the starch and soybean powder was shown as where, Y was the ESP production and A and B were starch and soybean powder, respectively. The model terms with “Prob > F” less than 0.05 are regarded significant.

*p* = 31.58416–4.40242 × A+0.08066B−0.02187 × A × B + 0.2044 × A^2^+0.010267 × B^2^.

### 3.3 Verification of optimum condition

**FIGURE 3:**
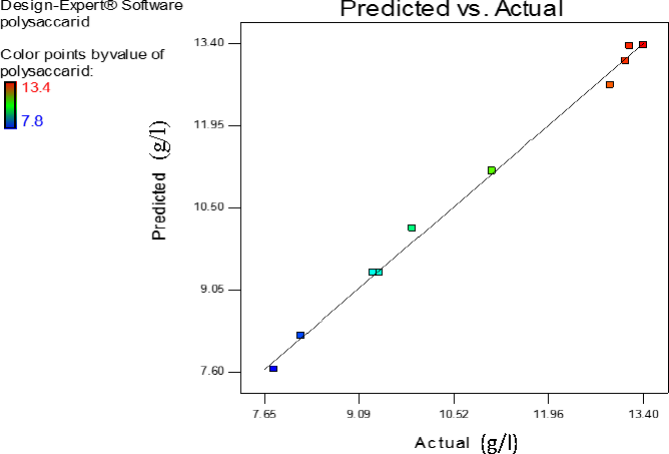
The curve of predicted values against actual values of the response

**FIGURE 4:**
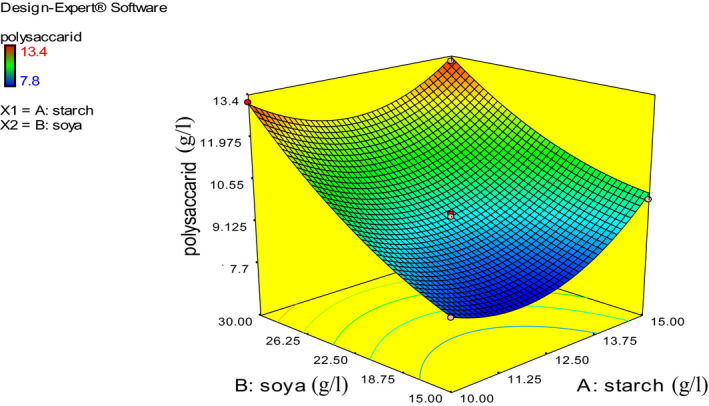
Response surface curve for CRMEP production by *Rhodotorula minuta* ATCC 10658 as a function of soybean and starch

There was no considerable difference between the predicted values and actual values of response, reflecting the adequacy of RSM. The experimental values (actual values) were compared with that of predicted values (Figure 3). On the basis of the findings of the present study, appropriate selections of the key substrates either in quality or quantity considerably enhanced CRMEP (Figure 4).

#### 3.3.1 CRMEP production

The 3‐dimensional plot showed that the highest CRMEP was attained at the lowest and highest quantity of starch combined with the highest soybean content. On the other hand, the result disclosed the negative effect of middle concentration of starch on CRMEP production. To be more precise, *R. minuta’* CRMEP was stimulated by the highest quantity of soybean powder. The same result was obtained by Moghannem, Farag, Shehab, & Azab, 2018 who stated that as carbon and protein source gradually climbed up to the highest points, EPS production sharply rose.

#### 3.3.2 Validation of the optimized culture conditions

Under the optimum conditions, the predicted CRMEP content reached the highest point (13.35 g/L). To validate the suitability of the model equation, predicted optimal condition was run. Under the actual experimental condition, the CRMEP level was 13.82 ± 0.2 g/L, which was slightly higher than the predicted maximum value (13.22 g/L).

### 3.4 Purification of CRMEP and production of RMEP

CRMEP was further purified using an anion‐exchange column of DEAE‐cellulose A52. The main fraction (RMEP) was collected and lyophilized for next analyses. RMEP, which turned a white powder, showed a negative response to the Bradford method and had no absorption at 280 and 260 nm in the UV spectrum, indicating the absence of protein and nucleic acid.

### 3.5 Methylation analysis of RMEP

Methylation analysis by GC–MS was employed to determine the types and proportions of glycosidic linkages of monosaccharide residues in RMEP. As summarized in Table 3, RMEP showed the presence of four components, namely 2,4,6‐Me_3_‐Man, 2,3,6‐Me_3_‐Man, 2,4‐Me_2_‐Man, and 2,3,4,6‐Me_4_‐Glc in molar ratios of 3.35:3.07:1.30:1.36 (about 3:3:1:1). This result revealed that Man and Glc accounted for about 85% and 15% of the total methylated residues, respectively. It was also revealed that some mannopyranosyl residues (around 17%) were branched and the molar number of 1,3,6‐linked Man*p* was approximately equal to the number of the 1‐linked Glc*p*, which implied all sugar residues appeared to have been completely methylated and the RMEP branches terminated with glucose residues. Moreover, the methoxyl groups were not observed at the C‐5 position, indicated that all the sugar residues existed in the pyranose ring forms. On the basis of the aforementioned results, it can be concluded that RMEP had a backbone chain of 1,3‐linked and 1,4‐linked Man*p* residues with side chains of terminal Glc*p* residues substituted in *O*‐6 position of some 1,3‐linked Man*p*.

**TABLE 3:** GC–MS data analysis of methylated RMEP

Methylated sugar	Molar ratio	Mass fragments (m/z)	Type of linkage
2,4,6‐Me_3_‐Man	3.35	43, 45, 87, 101, 118, 129, 161	→3)‐Man*p*‐(1→
2,3,6‐Me_3_‐Man	3.07	43, 45, 59, 87, 102, 113, 118, 129, 162, 233	→4)‐Man*p*‐(1→
2,4‐Me_2_‐Man	1.30	43,87,118,129,189	→3,6)‐Man*p*‐(1→
2,3,4,6‐Me_4_‐Glc	1.36	43,45,71,87,102,118,129,145,162,205	Glc*p*‐(1→

Our results were in accordance with that of Ramirez (2016), who reported that EPS, produced by *Rhodotorula minuta* BIOTECH 2,178, composed of mannose and glucose (Ramirez, 2016). The author gave no more information about molar ratios of the monosaccharides. Seveiri et al. (2019) studied monosaccharide composition of exopolysaccharides from *Rhodotorula minuta* IBRC‐M 30,135 (Seveiri et al., 2019). Their results showed the presence of glucose, mannose, and rhamnose in a molar ratio of about 3.8:3.0:1.0. The authors did not use progressive purification steps such as the ion‐exchange column chromatography used in this study.

## 4 CONCLUSION

In this study, one factor at a time was used to identify the key substrates with a great impact on exopolysaccharide production, named CRMEP. The highest content of CRMEP in starch media indicated that this substrate could be assimilated by *R. minuta* ATCC 10658. The highest content of protein level stimulated CRMEP production. CRMEP was chromatographed on a column of DEAE‐cellulose A52 for further purification and yielded RMEP. GC–MS results indicated that RMEP consisted of mannose and glucose at a molar ratio of about 1.4:7.7. Structural study revealed that RMEP possessed a backbone of → 3)‐Man*p*‐(1 → and →4)‐Man*p*‐(1 → residues, with branches attached to *O*‐6 of → 3)‐Man*p*‐(1→ (~17%) by Glc*p*‐(1→.

## CONFLICT OF INTEREST

The authors declare that they have no conflict of interest.
